# Epigenetic Regulation in the Pathogenesis of Sjögren Syndrome and Rheumatoid Arthritis

**DOI:** 10.3389/fgene.2019.01104

**Published:** 2019-11-13

**Authors:** José Santiago Ibáñez-Cabellos, Marta Seco-Cervera, Rebeca Osca-Verdegal, Federico V. Pallardó, José Luis García-Giménez

**Affiliations:** ^1^Center for Biomedical Network Research on Rare Diseases (CIBERER), Institute of Health Carlos III, Valencia, Spain; ^2^INCLIVA Health Research Institute, Mixed Unit for rare diseases INCLIVA-CIPF, Valencia, Spain; ^3^Department of Physiology, Faculty of Medicine and Dentistry, University of Valencia, Valencia, Spain

**Keywords:** epigenetics, autoimmune diseases, epigenetic pathways, rheumatic diseases, DNA methylation, histone modifications, miRNAs

## Abstract

Autoimmune rheumatic diseases, such as Sjögren syndrome (SS) and rheumatoid arthritis (RA), are characterized by chronic inflammation and autoimmunity, which cause joint tissue damage and destruction by triggering reduced mobility and debilitation in patients with these diseases. Initiation and maintenance of chronic inflammatory stages account for several mechanisms that involve immune cells as key players and the interaction of the immune cells with other tissues. Indeed, the overlapping of certain clinical and serologic manifestations between SS and RA may indicate that numerous immunologic-related mechanisms are involved in the physiopathology of both these diseases. It is widely accepted that epigenetic pathways play an essential role in the development and function of the immune system. Although many published studies have attempted to elucidate the relation between epigenetic modifications (e.g. DNA methylation, histone post-translational modifications, miRNAs) and autoimmune disorders, the contribution of epigenetic regulation to the pathogenesis of SS and RA is at present poorly understood. This review attempts to shed light from a critical point of view on the identification of the most relevant epigenetic mechanisms related to RA and SS by explaining intricate regulatory processes and phenotypic features of both autoimmune diseases. Moreover, we point out some epigenetic markers which can be used to monitor the inflammation status and the dysregulated immunity in SS and RA. Finally, we discuss the inconvenience of using epigenetic data obtained from bulk immune cell populations instead specific immune cell subpopulations.

More than 200 different disorders are considered autoimmune rheumatic diseases, but immune system complexity and the diversity of shared features make diagnosis and prognosis arduous tasks, especially in early follow-up stages (Alarcón et al., 1991). Studies performed using monozygotic twins have revealed an important epigenetic role in the progression of rheumatic diseases in addition to genetic factors (MacGregor et al., 2000;Javierre et al., 2010). Autoimmune rheumatic diseases, such as Sjögren syndrome (SS) and rheumatoid arthritis (RA), are characterized by pain and chronic joint inflammation. In fact, given the overlap of certain clinical features between SS and RA, it is assumed that the genetic contribution to SS and RA may be due to a multigenic contribution, which may affect many immunological-related mechanisms (**Table 1**). In line with this, Yang et al., suggested that patients with SS should be explored for a second autoimmune associated disease or more, such as RA (Yang et al., 2018), because all autoimmune diseases share a common phenotype and mechanisms. Therefore, genetic and epigenetic factors might influence poly-autoimmunity development (Anaya et al., 2016).

**Table 1:** Principal and common characteristics of RA and SS.

Disease	Prevalence (%)	General and common clinical features	Candidate genes	Organs and tissues affected	Autoantigens	Epigenetics mechanisms
**RA**	0.8	Female prevalence Pain and chronic joint inflammation Reduced mobility When a person suffers RA and SS, their course worsen and comorbidities and mortality increase Morning stiffness Interstitial lung disease Anemia Hyperinmmunoglobulinemia Xerophthalmia and xerostomia Rash Lymphoadenopathy Thyroid involvement (hypothyroidism) Hypertension	ANKRD55 CD247 HLA-DRB1 IL2RA IL2RB IL6 LACC1 MIF PTPN2 PTPN22 STAT4	Heart Bones Lung Synovial joint tissue Connective tissue	Rheumatoid factor Cyclic citrullinated peptides (anti-CCP) hnRNP-A2/RA33 Ro/SSA Sa 50 kDa protein Stress proteins (hsp60, BiP, hsp90) Glucose-6 phosphate isomerase Calpastatin	Hypomethylated *IFNG* promoter in pro-inflammatory T-cells (CD4^+^CD28T- T-cells) Hypomethylation is apparently accompanied by the hyperacetylation of histones, which contributes to the control of epigenetic programs in enhancer regions miR-146a and miR-155 appear as relevant epigenetic switches, and both can be considered to monitor inflammation status
**SS**	0.1–0.6		IRF5 STAT4 IL12A BLK CXCR5 TNIP1	Salivary glands Exocrine glands Lacrimal glands Connective tissue Lungs Bowel	Ro/SSA La/SSB Antinuclear antibodies (ANA) Rheumatoid factor Cryoglobulins Centromere (ACA) Cyclic citrullinated peptides (anti-CCP) Mitochondria (AMA) Muscarinic 3 receptor Carbonic anhydrases Smooth muscle

He et al. described RA as a systemic autoimmune disease that causes extra-articular complications and damage (He et al., 2013a). SS is defined as a chronic autoimmune disease caused by the diminished function of salivary and lacrimal glands due to the lymphocytic infiltration of exocrine glands (Ramos-Casals et al., 2007;He et al., 2013a). SS can be classified as primary or secondary, depending if it coexists with other autoimmune diseases or not, such as RA (Ramos-Casals et al., 2007). Both SS and RA present female predominance and produce chronic inflammation of joints which, in turn, causes pain and impedes normal mobility in affected patients. It is well-known that a person who suffers both pathologies, e.g. secondary SS and RA, has a worse prognosis and faces more comorbidities and increased mortality (He et al., 2013a).

Of the common features found in both RA and SS, we underscore chronic inflammation, the interaction of the immune system and other tissues, such as skeletal tissue in RA and exocrine glands in SS, and autoimmunity that produce tissue damage and destruction which, in turn, lead to reduced mobility. Initiation and maintenance of the chronic inflammatory stages accounts for several mechanisms involving immune cells as key players. Pro-inflammatory cytokines, such as interleukin-1 (IL-1), IL-6, IL-8, and tumor necrosis factor α (TNF-α), can be produced by circulating monocytes that differentiate to macrophages or dendritic cells (Arango Duque and Descoteaux, 2014). Monocytes produce reactive oxygen species (ROS) and cyclooxygenase-2 (COX2) as mediators of inflammation (Lu and Wahl, 2005), and attract T- and B-cell chemokines which, in turn, produce pro-inflammatory cytokines. Activated B-cells are able to present autoantigens and generate autoantibodies that preserve inflammation and can, consequently, lead to tissue destruction in these autoimmune rheumatic diseases. In fact, a hallmark of autoimmune rheumatic diseases is the presence of autoantibodies at non organ-specific antigens, specifically antigens occurring in nucleated cells or among circulating plasma proteins (Aggarwal, 2014). Diversity in environmental stimuli requires a quick dynamic response of immune cells. In this fast efficient functional response, epigenetic mechanisms play an essential role in, for example, hematopoietic cell differentiation. Relevant differences in epigenetic regulation between innate and adaptive immune cells have been described. Most are implicated in the hematopoietic process, although other epigenetic alterations are associated with myeloid and lymphoid lineage function (Avgustinova and Benitah, 2016). Inflammation as a transitory physiological process protects from pathogenic invasions, while inflammation malfunction can produce tissue damage and organ dysfunction, and can mediate several pathological processes. Genetic and epigenetic variability in physiological and pathological processes in immune cells is a complex and carefully regulated equilibrium that is difficult to decipher.

This review shows the epigenetic mechanisms related to RA and SS so as to identify the unclear association between SS and RA by elucidating the intricate mechanisms underlying the phenotypic features of both autoimmune diseases.

## Epigenetic Regulation of Genetic Programs in Immune Cells

Genome-wide associated studies (GWAS) have identified hundreds of risk loci for autoimmunity (Parkes et al., 2013). Integrated genetic and epigenetic fine mapping may help to identify the causal variants and epigenetic drivers that lead to autoimmune disease-associated loci and, in turn, contribute to explore their role in the control of the immune response (Farh et al., 2015).

Mammals' blood system have highly specialized cell types that derive from hematopoietic stem cells (HSCs). HSCs play the important role of maintaining the homeostasis of specialized cell types' production and self-renewal (Wilson et al., 2008, Wilson et al., 2009). Epigenetic studies have demonstrated that during the differentiation of HSCs, DNA methylation, microARNs (miRNAs), and histone modifications play key roles in expression programs by shifting from pluripotency and proliferation to mature differentiated immune cells (Avgustinova and Benitah, 2016; Mehta and Baltimore, 2016). Some of these mechanisms can fail due to erroneous epigenetic program, which may alter the immune responses in SS and RA. The next part of this review describes some of epigenetic drivers that lead to autoimmunity and altered immune response.

### DNA Methylation

DNA methyltransferases and the ten-eleven translocation (TET) families of enzymes regulate DNA methylation and demethylation during hematopoietic differentiation. For this reason, whole genome bisulfite sequencing (WGBS) has been performed to clarify DNA methylation changes in immune cell development (Morales-Nebreda et al., 2019). For example, Cabezas-Wallscheid et al. compared gene expression profiles and differentially methylated regions (DMRs) between HSCs and four multipotent progenitor populations (MPP) in mice. During an early transition of HSC to MPP, they found an association between DMR and inversely correlated gene expression during differentiation. In the most inversely correlated genes, they observed genes related to hematopoietic differentiation and HSCs function, such as *Hoxb2*, *Rorc*, and *Cd34* (Cabezas-Wallscheid et al., 2014). Differences in the methylation profile have been described as being lineage-specific in innate and adaptive immune systems. HSCs differentiate to multipotent progenitors of lymphoid (CLP) and myeloid (CMP) lineages, which present different DNA methylation patterns. In particular, CLP show increased DNA methylation in a diversity of transcription factors that regulates myeloid differentiation (e.g. *Gata2*, *Tal1*, and *Lmo2*) (Bock et al., 2012).

Both B- and T-cell activation, and proliferation, differentiation, and plasticity, are regulated and mediated by not only epigenetic mechanisms, but also by transcription factors and signaling molecules. The combinational activity of these mechanisms produces a characteristic transcriptome to each subset of differentiated B- and T-cells. The transition from naive to effector B-cells and T-cells exhibits different DNA methylation patterns. In fact, a DNA methylation analysis of B-cells performed in different development stages has shown a hypomethylation tendency after activation, which is reverted in memory B-cells. In addition, DNA methyltransferases (i.e. DNMTs; DNMT1, DNM3A, and DNMT3A) have also shown a shift in their expression levels between naive B-cells and late-stage differentiated B-cells (Lai et al., 2013). Activation of the axis JAK3-STAT6 pathway by IL-4 produces demethylation, which is TET2-dependent in monocytes and necessary for acquiring proper dendritic cell and macrophage identity (Vento-Tormo et al., 2016).

The DNA methylation regulation of T-lymphocyte differentiation has been widely described (Kanno et al., 2012; Morales-Nebreda et al., 2019) and is briefly described herein. The development of CD4^+^ helper T-cells and CD8^+^ cytotoxic T-cells from CLP is a complex regulated process in which methylation plays an important role. For example, Sellars et al. found that double negative (CD4^−^CD8^−^), double positive (CD4^+^CD8^+^) timocytes, and mature CD8^+^ cytotoxic T-cells present hypermethylation in a DMR near the *Cd4* gene, while CD4^+^ helper T-cells showed hypomethylation. These authors also revealed that DNMT1 is responsible for the *Cd4* gene silencing in CD8^+^ cytotoxic T-cells (Sellars et al., 2015). Activated CD4^+^ helper T-cells are guided to proliferate and differentiate to diverse T-helper cells to perform distinct functions. Many conditional deletion models of different DNMTs have pointed out the importance of methylation in regulating cytokine expression and the stabilization of subset IFN-γ-producing Th1 and IL-4-producing Th1 cell phenotypes (Lee et al., 2001;Gamper et al., 2009). Furthermore, the DNA methylation of many cys-regulatory elements involved in *lfng* expression in mice becomes demethylated in the IFN-γ-producing subset of Th1 cells, although these regions remain methylated in the subset IL-4-producing Th2 cells (Schoenborn et al., 2007). Another example of the importance of DNA methylation in the regulation of differentiation to various subsets of T-helper cells is regulatory T cells (Treg), which are characterized by Foxp3 expression (Hori et al., 2003). Lal et al. have described demethylated levels of a specific upstream *Foxp3* CpG site in the *Foxp3* enhancer of naive Treg compared to CD4^+^ helper T-cells and TGFβ-induced peripheral Treg (Lal et al., 2009). Finally, a recent WGBS study has identified up to 339 DMRs in Treg cells that map to the sites encoding for *Ctla4*, a gene that transmits an inhibitory signal to T-cells (Delacher et al., 2017).

### miRNA Regulation

miRNAs are ≈22 nucleotides noncoding RNAs that post-transcriptionally regulate the gene expression of their mRNA targets by binding to their 3'UTR. In the last decade, increasing knowledge about the role of the miRNAs that control specific transcriptomic programs has demonstrated the relevance of these epigenetic changes during the differentiation, development, and maturation of immune cells (Bissels et al., 2012; Mehta and Baltimore, 2016). For example, the differential expression of miR-155, let-7 and miR-21 may be implicated in altered long-term HSCs proliferation (Gruber et al., 2009). The differential expression of cluster miR-99b-let-7e-miR-125a enhances long-term HSCs self-renewal by conferring protection from apoptosis thanks to BCL-2 homologous antagonist/killer 1 (BAK1) being targeted by miR-125a (Guo et al., 2010). The expression of cluster miR-99a-let-7c-miR-125b promotes HSC differentiation by targeting TGF-ß signaling activators and WNT signaling repressors (Emmrich et al., 2014). In addition, TLR stimulation induces nuclear factor κß (NF-κß) that stimulates inflammamiR miR-146a which, in turn, is a TLR and cytokine signaling negative regulator by targeting TNF receptor-associated factor 6 (TRAF6) and interleukin receptor-associated kinase 1/2 (IRAK1/2) (Taganov et al., 2006). It is noteworthy that the polymorphisms in IRAK1/2 have been considered risk factors for autoimmune diseases, such as RA (Atabaki et al., 2017) and SS. Furthermore, the downregulation of miR-146 brings about the increased secretion of cytokines from hematopoietic progenitors (Zhao et al., 2014), and a polymorphism in the 3'-UTR of IRAK1 in the recognition region of miR-146a, which has been shown to be a factor of susceptibility of RA (Chatzikyriakidou et al., 2010).

The regulation of diverse innate immune cells in several differentiation stages has been reported (Kumar Kingsley and Vishnu Bhat, 2017). Macrophage development and immunity regulation as act as principal players miR-146a and miR-155 (Taganov et al., 2006; O'Connell et al., 2007;Ghani et al., 2011). The development of natural killer cells (NK) to natural killer T-cells (NKT) is controlled by miR-150 (Bezman et al., 2011). Granulocytic differentiation is regulated by the levels of miR-223 whose promoter is, in turn, regulated by either nuclear factor 1 A-type (NFI-A) or CCAAT/enhancer-binding protein-α (C/EBPα) binding (Fazi et al., 2005).

Lots of reviews have described the important role of miRNAs in adaptive immunity (Dooley et al., 2013; Marques et al., 2015; Mehta and Baltimore, 2016; Zhang et al., 2018). miRNAs play an essential role in B-cell development and function, and one example of such is miR-181a-5p (Georgantas et al., 2007; Jensen et al., 2013). Other examples include "inflamma-miRs" miR-155, miR-146a, and miR-150, which are key regulators of the B-cell function. The germinal center response involves miR-155 as an essential player for B-cell maturation and proliferation (Vigorito et al., 2013) and miR-146 deletion produces autoantibodies from B-cells (Boldin et al., 2011). Low expression miR-150 levels in pro-B cells inhibits the progression of pre-B cells differentiation in bone marrow, probably by the repression of its target gene, the MYB transcription factor (Xiao et al., 2007).

As in B-cell development and function, diverse and cell‐specific expression patterns of miRNAs have been described in T-cell subsets (Monticelli et al., 2005; Wu et al., 2007; Sethi et al., 2013). Some relevant miRNAs found in lymphoid cell lines are miR-150, miR-155, miR-146a, and cluster miR-17-92. miR-150 controls early T-cell development inhibition *via* the downregulation of Notch (Ghisi et al., 2011). miR-155 inhibits Th2 cell proliferation by targeting c-Maf, which is a transactivator of IL-4 (Rodriguez et al., 2007). At the same time, miR-155 is able to lead the differentiation of the Th1 response (Banerjee et al., 2010) and plays a special role regulating the differentiation of Treg and T-helper 17 cells by targeting *SOCS1* and controlling JAK/STAT signaling (Yao et al., 2012). Guerau-de-Arellano et al. have described how miR-128 and miR-340 are both implicated in the inhibition of Th2 cell lineage by directing differentiation to Th1 cells (Guerau-de-Arellano et al., 2011). miR-126 has been correlated with the promotion of Th2 cells (Mattes et al., 2009) and there are reports that miR-17-92 cluster deficiency reduces T-bet and IFN-γ expression, which are characteristic of Th1 lineage, and promotes Treg cell differentiation (Jiang et al., 2011). Here it is stressed that T-bet plays key role in autoimmunity development. Finally, another important miRNA in different subsets of T-cells is miR-146a, whose increased expression has been observed in effector Th1 (Monticelli et al., 2005) and Treg (Cobb et al., 2006), but not in Th2 cells and naive CD4^+^ T cells (Monticelli et al., 2005). Furthermore, as in myeloid cells, miR-146a regulates NF-kß signaling (Taganov et al., 2006) and targets STAT1 to thus control the Treg function (Lu et al., 2010).

### Histone Modification

The conformational chromatin changes produced by histone post-translational modifications (PTMs) (e.g., acetylation, methylation, phosphorylation, etc.) could result in easy or blocked DNA accessibility. These reversible modifications have different consequences depending on the affected histone protein or specific residue, which is known as the histone code. Generally speaking, acetylation of histone lysine residues (H3K9, H3K14, H4K5, and H4K16) and methylation (H2BK5, H3K4, H3K36, and H3K79); phosphorylation of histone H3 threonine 3 (H3T3) and serine (H3S10 and H3S28), as well as of histone 4 serine 1 (H4S1) and H2BK120 ubiquitinylation, are marks of open chromatin that increase DNA accessibility to transcription factors and produce increased gene expression. On the contrary, other marks, like the methylation (specifically di-methylation and tri-methylation) of H3K9, H3K27, and H4K20, the ubiquitination of H2AK119, and the sumoylation of H2AK126, H2BK6, and H2BK7, are associated with compacted chromatin and gene repression. In contrast to DNA methylation and miRNA regulation, fewer studies have been conducted into histone modifications in immune cells because of their relative complexity and the difficulty to transfer them to clinical practice. However, alterations of different histone PTMs, in polycomb repressive complexes, and also in histone modifying enzymes, have been described in immune system development and immune disorders (Loizou et al., 2009; Wilting et al., 2010;He et al., 2013b; Vidal and Starowicz, 2017; Di Carlo et al., 2018). Chen and coworkers described how the histone H3 lysine 79 di/trimethylated (H3K79me2/3) modifications by the disruptor of telomeric silencing-1-like (Dot1l) methyltransferase at the *Il6* and *ifnb1* promoters are abundant in active macrophages (Chen et al., 2018). Kdm6a demethylase promotes *Il6* and *ifnb1* overexpression in primary macrophages by lowering the H3K27me2/3 levels at their promoters (Li et al., 2017b). B-cell activation increases the levels of H3K4me3, H3K9ac, and H3K14ac in the promoter region of activation-induced cytidine deaminase enzyme (AID) by inducing the DNA cleavage of the regions in the immunoglobulin heavy chain (IgH) locus during class-switch recombination (CSR) (Chowdhury et al., 2008). H3K4me3, H3K9ac, H3K14ac, and H4K8ac are apparently essential for somatic hypermutation in AID target loci (Begum et al., 2012). Increased marks of H3K4me3 have been found in the *Ifng* promoter in Th1 cells and in the *Il4* promoter in Th2 cells, which are characteristic of each lineage, while consistently increased repressive marks of H3K27me3 have been found in both the *Ifng* promoter in T-helper Th2 cells and the *Il4* promoter in T-helper Th1 cells (Wei et al., 2009). In CD8^+^ cytotoxic T-cells, the *Ifng* promoter displayed decreased H3K27me3 levels compared to naive CD8^+^ T-cells, but H3K4me3 and H3K9ac increased, which are active epigenetic marks (Denton et al., 2011).

In a study performed in 21 autoimmune diseases, genetic and epigenetic data were combined to obtain a more comprehensive view of the etiology of these autoimmune diseases. By using chromatin immunoprecipitation (ChIP) data for H3K27ac, a mark of active enhancers and promoters, the authors elucidated the role of the SNPs detected in the genetic analysis by combining the results with those obtained in the ChIP study, and were able to link disease-associated SNPs with active enhancers (Farh et al., 2015). This example illustrates the utility of histone modification information for clinical research, specifically to know how the study of epigenetic regulation in rare autoimmune-related disorders can help us to completely elucidate the intricate and complex landscape of epigenetic control in the immune system. Thus, we herein describe how epigenetic alterations are linked to SS and RA in an attempt to identify special the relevant epigenetic mechanisms underlying the physiopathology of these autoimmune diseases (**Table 2**).

**Table 2:** Epigenetic Changes in RA and SS.

Disease	DNA methylation	Histone PTMs	miRNAs
**RA**	↓ RASFs, PBMCs and T cell (Corvetta et al., 1991; Karouzakis et al., 2009; Liu et al., 2011; Richardson et al., 1990) ↓ Synoviocytes (Nakano et al., 2013) ↓ IL6R, CAPN8 and HOXA11 genes in RASFs and ↑ DPP4 and HOXC4 in RASFs (de la Rica et al., 2013). ↓ IL-6 promoter in PBMCs (Ishida et al., 2012). ↑ IL-10 promoter in PBMCs (Fu et al., 2011). ↓ CXCL12 promoter in RASFs (Karouzakis et al., 2011). ↑ DUSP22 gene and ↓ GALNT9 gene in T cells (Glossop et al., 2014). ↑ CTLA-4 promoter in Treg cells (Cribbs et al., 2014.) ↑ EBF3 and IRX1 genes in RASFs (Park et al., 2013).	↑ H3 acetylation in synovial fibroblasts (Wada et al., 2014) ↑ HDAC1 levels in synovial tissues (Hawtree et al., 2015; Horiuchi et al., 2009; Kawabata et al., 2010) ↑ HDAC3 in fibroblast-like synoviocytes (Angiolilli et al., 2017) ↑ HDAC activity in synovial tissue (Huber et al., 2007)	↑ miR-24, miR-26a, and miR-125a-5p in plasma (Murata et al., 2013). ↑ miRNA-146a and ↓ miR-363 and miR-498 in CD4^+^ T cells (Li et al., 2010). ↑ miR-146a in PBMCs and synovium (Niimoto et al., 2010). ↑ miR-155 in PBMCs (Li et al., 2013). ↓ miR-146a and miR-155 in Treg cells (Zhou et al., 2015). ↑ miR-346 in RASFs (Semaan et al., 2011). ↑ miR-18a in RASFs (Trenkmann et al., 2013). ↓ miR-21-5p in Treg cells of RA (Dong et al., 2014). ↓ miR-23b in RASFs (Zhu et al., 2012). ↑ miR-126a in CD4^+^ T cells (Yang et al., 2015).
**SS**	↓ Type I IFN pathway genes promoter in CD19^+^ B cells (Imgenberg-Kreuz et al., 2016) ↓ CD70 promoter in CD4^+^ T cells (Yin et al., 2010) ↑ FOXP3 promoter in CD4^+^ T cells (Yu et al., 2013) ↓ STAT1, IFI44L, USP18, and IFITM1 promoter in naive CD4^+^ T cells (Altorok et al., 2014) ↓ PSMB8 and TAP1 promoter in labial salivary gland (Cole et al., 2016) ↓ KRT19 promoter in minor salivary glands (Konsta et al., 2016b, 2016a) ↓ global DNA methylation in SGECs (Thabet et al., 2013)	↑ H3K27ac in promoters and H3K36me3 in enhancers of B cells and monocytes (Konsta et al., 2015) ↑ H3K4me1, H3K36me3 and H3K27ac at enhancer regions in PBMCs (Imgenberg-Kreuz et al., 2016)	↑ miR-146a/b in PBMCs (Zilahi et al., 2012) ↑ miR-146 and ↓ miR-155 in PBMCs (Shi et al., 2014) ↓ miR-17, miR-18a, miR-19a, miR-20a, miR-19b-1, and miR-92a-1 in salivary glands (Alevizos and Illei, 2010) ↑ miR-34b-3p, miR-4701-5p, miR-609, miR-300, miR-3162-3p, and miR-877-3p in monocytes (Williams et al., 2016) ↑ miR-16a in labial salivary gland (Wang et al., 2018) ↑ miR-155-5 p, miR-222-3p, miR-146a-5p in CD4^+^ T and ↑ miR-28-5p in B lymphocytes (Wang-Renault et al., 2018) ↓ let-7d-3p, miR-30c-5p, miR-378a-3p and in CD4^+^ T and ↓ miR-378a-3p, miR-26a-5p, miR-30b-5p and miR-19b-3p in B lymphocytes (Wang-Renault et al., 2018) ↑ miR-181a-5p in PBMCs and SGECs (Peng et al., 2014; Wang et al., 2018) ↑ miR-155 in PBMCs and SGECs (Le Dantec et al., 2012; Pauley et al., 2011)

*↑* means that it is up-regulated. *↓* means that it is down-regulated.

## Sjögren Syndrome

Sjögren syndrome (SS; Disease identification number: OMIM:270150 and ORPHA:289390) is a chronic autoimmune disorder considered a rare disease, characterized by salivary and lacrimal glands being affected, which lead to dry eyes and dry mouth, expose the oral cavity to infections, caries, candidiasis, and imply musculoskeletal pain. SS may occur alone as primary SS or as secondary SS in association with other autoimmune diseases like systemic lupus erythematosus (SLE) or RA. Primary SS prognosis is not favorable and is linked to the onset of respiratory or kidney failure. However, secondary SS is characterized by keratoconjunctivitis and xerostomia, but is always associated with other autoimmune disorders. Therefore, prognosis depends strictly on the primary autoimmune pathology (He et al., 2013a). The problem lies in the presence of the lymphocytic infiltration of exocrine glands and other organs, together with the presence of various autoantibodies [reviewed in (Kapsogeorgou and Tzioufas, 2016); **Table 1**]. Patients with SS are at higher risk of developing lymphoma and primary liver cirrhosis and can, in some cases, develop non-Hodgkin's lymphoma (Pagano et al., 2013; Ciechomska and O'Reilly, 2016; Vivino, 2017). The exact cause of SS is unclear, but several lines of evidence suggest a wide array of mechanisms including the dysregulation of the immune system which can destroy exocrine glands, as consequence of the production of chemokines by epithelial cells which attract T cells and dendritic cells producing an unappropriated immune response in the acinar and ductal epithelial cells (Nordmark et al., 2006). Moreover, genetic susceptibility and the contribution of epigenetic factors in the SS pathophysiology have been also proposed. Genes *IRF5* (interferon regulatory factor 5), *CXCR5* (C-X-C chemokine receptor type 5), *TNIP1* (TNFAIP3-interacting protein 1), *IL12A* (interleukin-12 subunit alpha), *BLK* (B lymphoid tyrosine kinase), and *STAT4* (signal transducer and activator of transcription 4) have been proposed in several studies as gene candidates for SS susceptibility (Lessard et al., 2013;Burbelo et al., 2014). Genome-wide and epigenome-wide association studies have been performed to identify the genes and epigenetic marks involved in SS (Low and Witte, 2011; Konsta et al., 2014).

**Figure 1:**
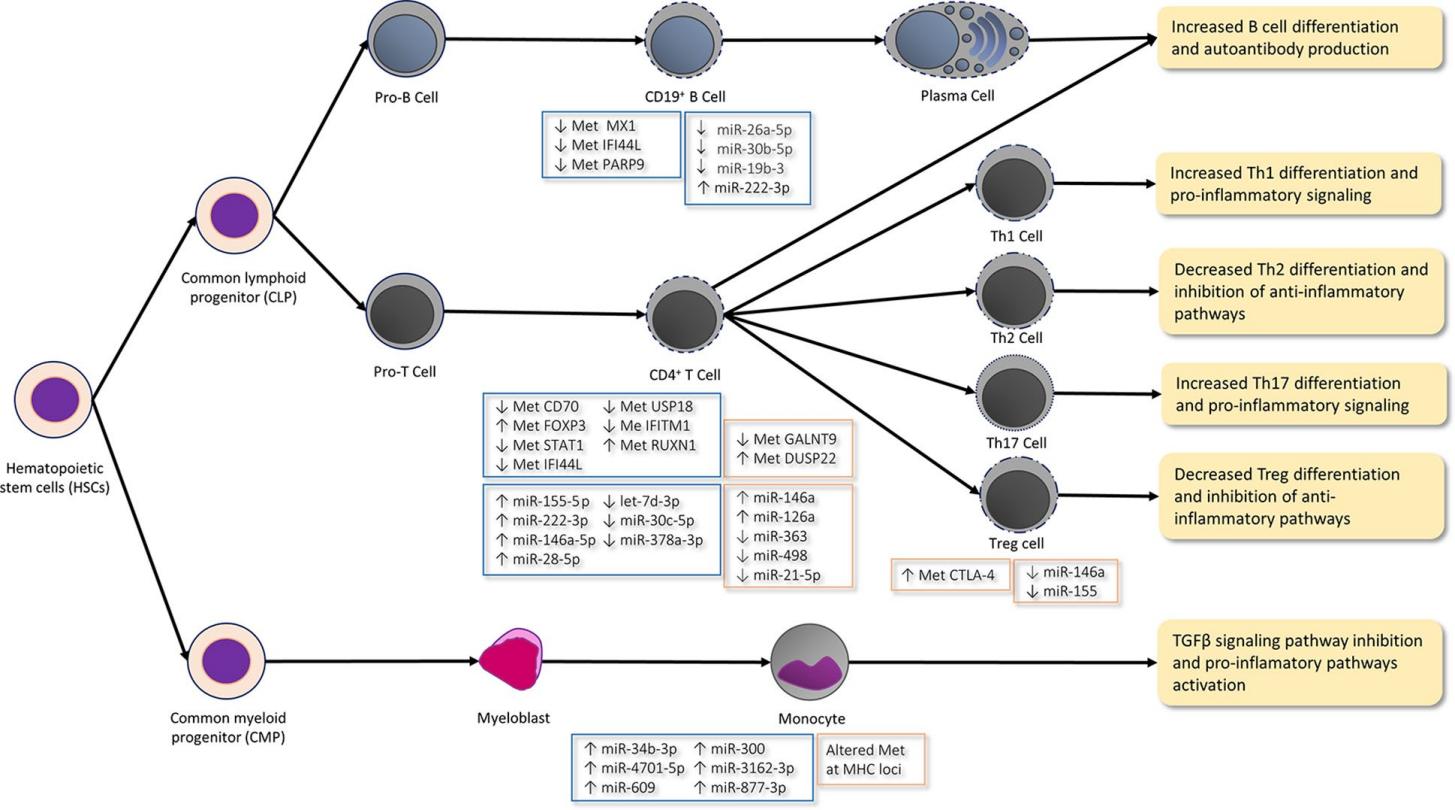
Epigenetic mechanisms in immune cells differentiation and function in arthritis rheumatoid (AR; orange boxes) and Sjögren syndrome (SS; blue boxes). In the CD19^+^ B-cells of SS patients, type I IFN genes, such as genes MX1, IFI44L, and PARP9, are hypomethylated (↓ Met), which agrees with the hypomethylation of some other IFN-γ-regulated genes (e.g. STAT1, IFI44L, USP18, and IFITM1) in CD4^+^ T-cells and with the IFN response activation observed in these patients. Moreover, low levels (↓) of miR-26a-5p, miR-30b-5p, and miR-19b-3p and high miR-222-3p levels (↑) are reported in the CD19^+^ B-cells of SS patients. Low levels miR-30b-5p bring about an increase of the B-cell activating factor (BAFF) and autoantibody production. The hypomethylation of the CD70 promoter in the CD4^+^ T-cells of SS patients leads to the increased expression of the CD70 gene, which interacts with CD27 during B- and T-cell contact by promoting plasma cell differentiation and IgG production. The hypermethylation (↑ Met) of the RUNX1 gene in SS regulates the maturation of hematopoietic stem cells. The high expression of inflamma-miRs miR-155-5 p, miR-222-3p, and miR-146a-5p and the low expression of let-7d-3p, miR-30c-5p, and miR-378a-3p are described in CD4^+^ T-cells. ImmflamamiRs miR-155 and mir-146a inhibit Th2 proliferation and promote the Th1 response. Furthermore, miR-155 also promotes the differentiation and function of Th17 and Treg. The hypermethylation of the FOXP3 promoter lowers the FOXP3 expression in the CD4^+^ T-cells of SS and is characteristic of the differentiated and functional Treg. The hypermethylation of the DUSP22 gene and the hypomethylation of the GALNT9 gene in CD4^+^ T-cells are implicated in the IL-6/STAT3-mediated signaling pathway by contributing to autoimmunity and promoting the pro-inflammatory Th17 cells differentiation in RA patients. High inflamma-miR miRNA-146a levels and low miR-363, miR-21-5, and miR-498 levels are reported in the CD4^+^ T-cells of RA patients. Low miR-21-5p levels promote Th17 cell differentiation, while suppressing Treg development. Furthermore, high miR-126a levels inhibit DNM1I which, in turn, produces the hypomethylation of CD11a and CD70 by increasing their expression and promoting the autoimmune response. The methylation of the CTLA4 promoter inhibits Treg activity in RA. In contrast to the miRNA levels observed in the CD4^+^ T-cells of RA patients, miR-146a and miR-155 are lower in Treg cells after T-cell stimulation. miR-146a regulates NF-kß signaling and targets STAT1 by thus controlling the Treg function. Hence, the low levels of this miRNA bring about the inhibition of Treg differentiation and function. The differential methylated sites located in the MHC region are described in the CD14^+^ monocytes of RA patients. Moreover, the levels of miR-34b-3p, miR-4701-5p, miR-609, miR-300, miR-3162-3p, and miR-877-3p are higher in the monocytes of SS patients, which may inhibit the TGFβ signaling pathway. Thus, an unbalanced differentiation takes place from the CD4^+^ T-cells to the Th1 and Th17 cells, which promotes pro-inflammatory pathways and increases autoantibody production in both diseases.

The DNA methylation of immune cells is a key regulatory mechanism that underlies SS. Less global DNA methylation in salivary gland epithelial cells (SGECs) has been observed and linked to decreased DNMT1 and increased GADD45α (Thabet et al., 2013). The DNA methylation levels of SGECs have been inversely correlated with SS severity and B-lymphocyte infiltration. The global DNA methylation levels have been reported to increase in the SGECs of SS patients after administering the anti-CD20 monoclonal antibody rituximab (Devauchelle-Pensec et al., 2007; Thabet et al., 2013). The hypomethylation of the *CD70* promoter in CD4^+^ T-cells brings about *CD70* gene expression (Yin et al., 2010). CD70 is a B-cell costimulatory molecule that interacts with CD27 during B- and T-cell contact by promoting plasma cell differentiation and IgG production and, therefore, contributes to the immunological disarrangement and autoimmune response in SS. Otherwise, the hypermethylation of the *FOXP3* promoter leads to lower FOXP3 expression, mRNA, and protein levels in the CD4^+^ T-cells of SS (Yu et al., 2013). Low DNA methylation levels in minor salivary glands generate an increased *KRT19* gene expression by producing elevated levels of epithelial protein cytokeratin-19 (CK-19), while hypermethylation has been associated with low protein CK-19 levels. A therapy conducted with DNMT inhibitor 5-azacytidine has increased *KRT19* gene expression and CK-19 protein levels (Konsta et al., 2016a), which demonstrates that DNA methylation is a key player in the control of the expression of both genes. A recent genome-wide DNA methylation study conducted in CD19^+^ B-cells and minor salivary glands has identified prominent hypomethylation sites of interferon (INF)-regulated genes, such as *MX1*, *IFI44L*, and *PARP9*. The hypomethylation of ING-regulated genes in SS B-cells brought about an increase in their gene expression, although an increase in *OAS2* gene expression took place in minor salivary glands (Imgenberg-Kreuz et al., 2016). The hypomethylation of some other ING-regulated genes, such as *STAT1*, *IFI44L*, *USP18*, and *IFITM1*, has been reported (Altorok et al., 2014). The hypomethylation of these genes is congruent with the IFN response activation observed in SS patients. Altorok et al. have also reported that transcription factor gene *RUNX1* is hypermethylated in patients with SS and regulates the maturation of hematopoietic stem cells (Okuda et al., 2001; Altorok et al., 2014) (**Figure 1**). RUNX1 has been associated with cancer predisposition (Asou, 2003; Kundu et al., 2005; Schlegelberger and Heller, 2017), which suggests a possible connection to lymphoma predisposition in SS patients. A recent study by Cole et al. has demonstrated the hypomethylation of *PSMB8* and *TAP1*, which increased the frequency of antigen-presenting cells in labial salivary gland tissues of SS patients (Cole et al., 2016). Moreover, the hypomethylation of *SSB* gene promoter 1 has been observed in the SGECs of SS patients with SSB/La autoantibodies compared to those from anti-SSB/La-negative patients (Konsta et al., 2016b).

miRNAs are dysregulated in SS. A reduction in cluster miR-17-92 (miR-17, miR-18a, miR-19a, miR-20a, miR-19b-1, and miR-92a-1) has been observed in the salivary glands of patients with SS (Alevizos and Illei, 2010). This cluster is involved in cell cycle, apoptosis, immune response, and other relevant cellular processes (Mogilyansky and Rigoutsos, 2013). As previously described, this cluster is relevant in lymphoid cell lines as it activates the characteristic expression programs of the Th1 lineage (i.e. T-bet and IFN-γ) and promotes Treg cell differentiation. Another study has observed increased levels of miR-146a, which regulates the innate immune and inflammatory response by producing the repression of *IRAK1* and increased *TRAF6* gene expression which, in turn, promote the NF-κB target genes expression in the peripheral mononuclear cells of SS patients (Zilahi et al., 2012). Conversely, Williams et al. have reported that miR-34b-3p, miR-4701-5p, miR-609, miR-300, miR-3162-3p, and miR-877-3p increase in SS monocytes (**Figure 1**). This miRNA profile revealed the broadest coverage of the predicted targets in the canonical TGFβ signaling pathway. Thus, their high levels may suppress this signaling pathway and direct the balance to the pro-inflammatory interleukin-12 and Toll-like receptor/NF-κB pathways (Williams et al., 2016). A recent study has detected the high expression of inflamma-miRs miR-155-5 p, miR-222-3p, miR-146a-5p, and miR-28-5p in CD4^+^ T-cells and miR-222-3p CD19^+^ B-cells, and the low expression of let-7d-3p, miR-30c-5p, and miR-378a-3p in CD4^+^ T-cells, and of miR-26a-5p, miR-30b-5p, and miR-19b-3p in CD19^+^ B-cells (Wang-Renault et al., 2018). As mentioned above, inflamma-miRs miR-155 and mir-146a inhibit Th2 proliferation and promote the Th1 response (**Figure 1**). Furthermore, miR-155 also promotes the differentiation and function of Th17 and Treg differentiation (Monticelli et al., 2005;Cobb et al., 2006; Taganov et al., 2006; Rodriguez et al., 2007; Banerjee et al., 2010; Yao et al., 2012). This result agrees with other studies, which have observed how miR-155 increases in the peripheral blood mononuclear cells (PBMCs) and SGECs of SS patients (Pauley et al., 2011; Le Dantec et al., 2012). Conversely, Shi et al. have noted high miR-146a levels and low miR-155 levels in the PBMCs of SS patients (Shi et al., 2014). It is worth mentioning that, as described in the Introduction, miR-146a and miR-155 are important regulators of B- and T-cell differentiation, proliferation and function, and both have been considered two key modulators in the control of the innate and adaptive immune response (Gottfried et al., 2012). Furthermore, low miR-30b-5p levels bring about an increase in the gene expression of the B-cell activating factor (BAFF) in the CD19+ cells of SS patients (Wang-Renault et al., 2018). High BAFF levels have been described in the serum, salivary gland B-cells, T-cells, and epithelial cells of these patients (Lavie et al., 2004;Daridon et al., 2007). In addition, these increased levels correlate with autoantibody production and, accordingly, mice models with high BAFF levels show phenotypic SS features (Mackay et al., 1999; Mariette, 2003).

Remarkably, increased levels of miR-181 and miR-16a in the labial salivary glands and PBMCs of SS patients are associated with the degree of inflammation in SS (Wang et al., 2018), which suggests that both miRNAs are biomarker candidates for monitoring the inflammatory course of SS. miR-181a-5p is associated with antigen sensitivity and the dysfunction of exocrine glands. Peng et al. have observed increased miR-181a-5p levels in the PBMCs of SS (Peng et al., 2014). This miRNA has been described to target the muscarinic receptor 3 gene (CHRM3)(Whisnant et al., 2013), whose variants have been associated with SS (Appel et al., 2011). Accordingly, autoantibodies against CHRM3 have been detected in the serum of SS patients (Gao et al., 2004). Other targets of miR-181a-5p are genes *TRIM21* and *SSB*, which encode for the 52kDa subunit of Ro/SSA ribonucleoprotein and the La/SSB ribonucleoprotein, respectively (Yang et al., 2016). SSA/Ro and SSB/La are regulated by miRNA let-7b, which is repressed in the SGECs of SS (Kapsogeorgou et al., 2011). Autoantibodies against these proteins have been detected in the serum, saliva, salivary gland epithelia, and B-cell infiltrating salivary glands of SS patients (Horsfall et al., 1989; Salomonsson and Wahren-Herlenius, 2003;Routsias and Tzioufas, 2007).

Finally, some authors have studied histone modifications at relevant genomic loci linked to SS risk variants to identify the critical role played by these variants in the disease. Active histone post-translational marks (e.g. H3K4me2, H3K4me3, and H3K9Ac) at the promoters of selected risk variants in B-cells have been observed in contrast to epithelial cells and monocytes. Active marker H3K27Ac and active marker H3K36me3 are enriched in B-cells and monocytes, in contrast to epithelial cells (Konsta et al., 2015). A recent study has reported an increase in the enhancer regions (H3K4me1 and H3K27ac) associated with the CpG sites hypomethylated in the whole blood of SS patients, while hypermethylated sites are underrepresented in these regions and also enriched for active post-translational mark H3K36me3 (Imgenberg-Kreuz et al., 2016). These results reveal that histones PTMs are also key components in the regulation of specific variants, and they also play a critical role in the genetic background and the risk of developing SS. Therefore, further studies should be undertaken to ascertain the role of histone PTMs in the regulation of the genetic risk factors linked to SS.

## Rheumatoid Arthritis

RA (Disease Identification Number: OMIM: 180300) is a systemic autoimmune inflammatory disease characterized by chronic joint inflammation and structural damage, with extra-articular manifestation, such as rheumatoid nodules, pulmonary involvement or vasculitis, and systemic comorbidities (Smolen et al., 2016). RA presents a large number of associated autoantibodies [reviewed in (Steiner, 2007) and (Conrad et al., 2010); **Table 1**]. The autoantibodies produced by the action of B- and T-cells in the synovial membrane promote the destruction of joints, suggesting a particular osteoimmunological interaction in RA (Okamoto and Takayanagi, 2011). Macrophages and granulocytes generate pro-inflammatory cytokines, chemokines, and ROS in synovial fluid by increasing the inflammatory response (Kinne et al., 2007). The origin of RA is unclear, but is considered to be a combination of genetic predisposition and epigenetic factors. Some of the genetic factors of RA are associated with the alleles of HLA-DR4, DR14, and some DR1 beta chains (Smolen et al., 2016), which are polymorphisms found in the gene of protein tyrosine phosphatase and nonreceptor type 22 (PTPN22) that regulate the activity of T- and B-lymphocytes, as well as the polymorphisms of the genes that regulate cytokine production (TNF and STAT4) (Conigliaro et al., 2017).

One of the most important epigenetic factors in RA is DNA methylation, which affects immune-related genes and, therefore, the immune response. Glossop et al. have observed differential DNA methylation in T- and B-cells, and also in synovial fibroblasts in early RA stages (Klein and Gay, 2015;Glossop et al., 2016). Global DNA hypomethylation has been found in rheumatic arthritis synovial fibroblasts (RASFs), PBMCs, and more specifically in the T-cells obtained from RA patients (Richardson et al., 1990;Corvetta et al., 1991; Karouzakis et al., 2009; Liu et al., 2011). The DNA methylation studies performed by Liu et al., by means of Infinium 450K methylation array (Illumina) from sorted CD14^+^ monocytes of RA patients and controls, found nine differential methylated sites located in the MHC region, which suggests that monocytes are more proximal to the pathogenic cell type (Liu et al., 2013). Here it is necessary to stress that the MHC class II gene expression in the macrophages that came from monocytes has been linked to RA progression (Mueller et al., 2007). Other authors have observed the hypomethylation of the pro-inflammatory cytokine *IL6* promoter and the hypermethylation of the anti-inflammatory *IL10* gene promoter in the PBMCs of RA patients (Fu et al., 2011; Ishida et al., 2012). Interestingly, reduced IL-10 production should contribute to the balance between CD4^+^ Th2 cells being lost to favor CD4^+^ Th1 cell function (Fiorentino et al., 1989; Said et al., 2010) (**Figure 1**). In line with this, IL-10 generally suppresses the proliferation and cytokine production of all T-cells, as well as the activity of macrophages. Despite the results of Fu et al. (Fu et al., 2011) showing the hypermethylation of the anti-inflammatory *IL10* gene in the PBMCs of RA patients, the results of Hernández-Bello et al. found an aberrant overexpression of the *IL10* gene in these patients (Hernández-Bello et al., 2017).

An alteration to the methylation of other genes has also been studied. Glossop et al. found the hypermethylation of the *DUSP22* gene and the hypomethylation of the *GALNT9* gene in the T-cells of RA (Glossop et al., 2014). *GALNT9* codifies for an important glycosyltransferase, which is essential in the first step mucin biosynthesis. Mucin is involved in RA (Ishino et al., 2010) and induces IL-6 expression in the PMBC of RA patients (Hamaguchi et al., 2011). *DUSP22* codifies for a tyrosine phosphatase that negatively regulates the IL-6/STAT3-mediated signaling pathway. It is noteworthy that IL-6 is a pivotal cytokine in RA. It contributes to autoimmunity (Fonseca et al., 2009) and promotes pro-inflammatory Th17 cells differentiation (Volpe et al., 2008; Yang et al., 2008). The methylation of the *CTLA*4 promoter inhibits the expression and activation of the indoleamine 2,3-dioxygenase pathway (IDO pathway) in the Treg cells of RA (Cribbs et al., 2014). This pathway is an important regulatory checkpoint that influences Treg activity by stabilizing and augmenting the immunosuppressive phenotype, and by preventing Treg reprogramming into nonsuppressive T helper-like cells (**Figure 1**). A genome-wide study has shown the hypomethylation of genes *IL6R*, *CAPN8,* and *HOXA11*, and the hypermethylation of *DPP4* and *HOXC4* in *RASFs* (de la Rica et al., 2013). The hypomethylation of the chemokine *CXCL12* promoter leads to an increase in matrix metalloproteinases which, in turn, cause the destruction of joints in RASFs (Karouzakis et al., 2011). The hypermethylation of genes *EBF3* and *IRX1* in RASFs has also been observed (Park et al., 2013). Nakano et al. presented a series of hypomethylated genes which clustered in the key pathways related to immune mechanisms, such as cell migration, focal adhesion, cell adhesion, trans-endothelial migration, and extracellular matrix interactions (Nakano et al., 2013). It is worth mentioning that DNA methylation dysregulation in RA could be a consequence of metabolic alterations. For example, increased levels of PMFBP1 (polyamine-modulated factor 1 binding protein 1) and SSAT1 (spermidine synthase) in RASFs promote polyamines metabolism, which may increase S-adenosyl methionine (SAM) consumption and, in turn, decrease the substrate required for DNA methylation in RASFs cells (Karouzakis et al., 2012). Some treatments used in RA patients, such as methotrexate (MTX), have been demonstrated to produce demethylation in *FOXP*3, which leads to the accumulation of Treg cells (Cribbs et al., 2015). Moreover, due to the changes in the methylation in *FOXP*3, Wieczorek et al. have suggested analyzing the methylation of *FOXP3* as a new method for counting Treg, evaluating disease status and identifying patients' responses to therapy (Wieczorek et al., 2009). Another study has shown that MTX can reverse the hypomethylated status and restore methylation levels once again by reducing the expression of the enzymes involved in demethylation in PBMC (de Andres et al., 2015).

More studies have demonstrated that miRNAs play an important role in RA. MiR-24, miR-26a, and miR-125a-5p are overrepresented in the plasma of RA patients, which suggests their feasible utility as biomarkers (Murata et al., 2013). Li et al. have observed an increase in miRNA-146a that correlated with TNF-α levels and with low levels of miR-363 and miR-498 in CD4^+^ T-cells (Li et al., 2010). Indeed as we indicate in the Introduction and describe in the SS section, miR-146a has been proposed as a key regulator of the immune system because it can control the secretion of cytokines, B-cell function, and NF-kß signaling, among others. Increased miR-146a levels are associated with IL-17 expression in the PBMCs and synovium of RA patients (Niimoto et al., 2010). In contrast, miR-146a and miR-155 lower in Treg cells after T-cell stimulation in RA patients (Zhou et al., 2015). miR-155 inhibits the transcript of SOCS1 and produces the upregulation of both TNF-α and interleukin IL-1β in the PBMCs of RA (Li et al., 2013). Other miRNAs have also been found to be involved in RA. For example, miR-346 controls TNF-ß synthesis in RASFs by tristetraprolin stabilization (Semaan et al., 2011). Tristetraprolin (also known as the zinc finger protein 36 homolog) is a protein that binds to AU-rich elements in the 3'-untranslated regions of the mRNAs of cytokines and promotes their degradation.

miR-18a plays an important role in the TNF-α-mediated signaling pathway through a feedback loop in the NF-κB signaling in RASFs (Trenkmann et al., 2013). Dong et al. have described a high Th17/Treg cells ratio in the PMBCs of RA patients and they have also found reduced expression levels in miR-21-5p in the CD4^+^ T-cells of these patients. Low miR-21-5p levels may promote Th17 cell differentiation while suppressing Treg cell development during chronic inflammation in RA (Dong et al., 2014) (**Figure 1**). Another study has reported that miR-23b show low levels in RASF. It also reported that this miRNA suppresses IL-17-associated autoimmune inflammation by targeting *TAB2*, *TAB3*, and *IKK*-α and that IL-17 is also a negative regulator of miR-23b expression (Zhu et al., 2012). This agrees with the increased IL-17 levels described in RA patients (Zhu et al., 2012), and also with a high ratio of Th17 cells (Dong et al., 2014), which are the main source of this cytokine. In addition, the increase in miR-126a promotes the inhibition of DNMTI, which produces the hypomethylation of the promoters of *CD11a* and *CD70* and, in turn, their expression increases in the CD4^+^ T-cells of RA (Yang et al., 2015). This mechanism may have similar consequences to those that occur in SS, where the hypomethylation of *CD70* in B-cells has been observed and, therefore, contributes to the autoimmune response.

Very few research studies have delved into the role of the PTMs of histones in specific gene or genomic regions in RA. Huber et al. have described an increase in the shifted balance of histone acetylase/histone deacetylase activity to hyperacetylation in the synovial tissue samples of RA patients (Huber et al., 2007). These results agree with increased IL-6 production in the RASFs of RA patients, where elevated H3ac and H3K4me3 levels were found in the proximal IL-6 promoter (Wada et al., 2014). Kawabata et al. have described increased histone deacetylase 1 (HDAC1) levels in the synovial tissues in RA patients, which have been correlated with a high cytoplasmic TNF-α concentration (Horiuchi et al., 2009; Kawabata et al., 2010). In agreement with the previous results, the inhibition of HDAC1 in RASFs and in a mouse collagen-induced arthritis model brought about changes in the expression of the genes related to proliferation, migration, and inflammation by, therefore, improving the RA phenotype (Hawtree et al., 2015). Furthermore, HDAC3 activity is necessary for type I interferon (IFNI) production and the subsequent activation of the signal transducer and activator of transcription 1 (STAT1) in fibroblast-like synoviocytes (FLS) (Angiolilli et al., 2017). In contrast, *HDAC5* expression is suppressed in RASFs by inflammatory cytokines, such as IL-1β and TNF-α, which produce interferon regulatory factor (IRF1) nuclear localization and the promotion of IRF1-regulated genes (Angiolilli et al., 2016). Finally, PADI4 is able to deiminate the arginine residues of histones by generating citrulline residues, and an increased PADI4 expression has been observed in RA synovial membranes (Chang et al., 2009).

## Conclusions

Recent research demonstrates the importance of epigenetics in the development of the most prevalent human autoimmune diseases because epigenetic dysregulation plays a relevant role in the pathophysiology of several autoimmune/inflammatory disorders, such as SS and RA (**Table 2**).

The increased expression of IFN-induced genes as a consequence of promoter hypomethylation has been demonstrated in the B-cells of SS patients. The hypomethylated *IFNG* promoter in pro-inflammatory T-cells (CD4^+^CD28T^−^ T-cells) has also been found in RA patients (Pieper et al., 2014). Hypomethylation is apparently accompanied by the hyperacetylation of histones, which may contribute to the control of epigenetic programs in enhancer regions. miR-146a and miR-155 appear as relevant epigenetic switches in both autoimmune disorders SS and RA. In particular, miR-146a is elevated in the PBMCs of both SS and RA (**Table 2**). This is particularly relevant because both miR-146a and miR-155 are recognized as "inflamma-miRs" in aging (Olivieri et al., 2013), a physiological process during which immunosenescence occurs (Fulop et al., 2018). Therefore, it is noteworthy that both miRNAs can be used to monitor not only the inflammation status in SS and RA, but also as markers of dysregulated immunity. Hence, many studies have aimed to elucidate age-dependent changes in chromatin accessibility, DNA methylation, and histone modifications in immune cells, particularly in T-cells (Tserel et al., 2015;Moskowitz et al., 2017; Cheung et al., 2018). The implication of these epigenetic regulation mechanisms in aging will improve our understanding of the phenotypical variations observed in disease progression of both RA and SS patients.

The miR-181 and miR-16a signature measured in the PBMCs of SS patients seems more specific. Both miRNAs are associated with the degree of inflammation (Wang et al., 2018), which indicates the role played by both miRNAs as biomarker candidates to monitor SS progression.

A large number of epigenetic studies in RA and SS have been conducted in PBMCs or affected tissues (RASF or salivary glands). Conversely, accumulating evidence in different subsets of immune cells reveals specific changes in the epigenetic marks that regulate the differentiation and function of each subset of cells (Bock et al., 2012; Sethi et al., 2013; Avgustinova and Benitah, 2016; Kumar Kingsley and Vishnu Bhat, 2017;Wu et al., 2018). Thus, it is reasonable to think that a more specific analysis of the epigenome in specific immune cell subsets should be carried out to unveil which type of immune cells is altered in these autoimmune diseases. This is an important fact because most epigenetic data have been obtained from bulk immune cell populations. However, in order to completely decipher the role of epigenetic mechanisms in autoimmune-related disorders, the whole epigenome needs to be elucidated. Single-cell technologies, such as mass cytometry or single-cell RNA sequencing, have revealed an unprecedented heterogeneity in different immune cell populations (Buenrostro et al., 2015; Cusanovich et al., 2015; Farlik et al., 2015). These strategies have allowed the whole methylome, chromatin regulation to be characterized by the ChIP-seq analysis, as well as the whole miRNome, which helps us to understand the pathogenesis of SS and RA by identifying biomarkers for disease management and predicting the long-term outcomes in these patients. For example, lymphoma is a long-term feature in about 5% of SS patients (Theander et al., 2006).

Furthermore, in order to understand the complexity of immune-related disorders, we must consider not only the complexity of immune cell regulation and epigenetic programs established in immune cell subpopulations, but also the interaction of immune cells with other tissues. The tissue environment can influence genetic programs in immune cells, and it is obvious that environmental factors may affect the inflammatory pathways in both RA and SS. For this reason, another issue to consider is how environmental factors affect the epigenetic regulation in autoimmune diseases. It is known that a narrow range of risk factors has been associated with rheumatic diseases, and also with epigenetic changes (Klareskog et al., 2006;Colafrancesco et al., 2016). In SS, factors such as stress, air pollution, vitamin D, or hormonal factors have been described as risk factors for this autoimmune disease to develop and progress (Colafrancesco et al., 2016). In RA, smoking is considered the commonest environmental risk factor to contribute to RA development and severity (Baka et al., 2009). Air pollution has been widely reported as an epigenetic modulator [see the review in (Li et al., 2017a)] and to increase the probability of developing systemic autoimmune rheumatic diseases such as SS (Bernatsky et al., 2016). Similarly, monocyte-specific smoking-associated DNA methylation patterns have been observed (Reynolds et al., 2017), which well matches an increased risk of RA development (Klareskog et al., 2011;Chang et al., 2014). Although many studies have related environmental factors to the development and/or progression of SS and RA, the actual mechanisms underlying these diseases are still unknown. Hence, epigenetic regulation could provide new pieces in the intricate puzzle of the environmental effects in SS and RA and, thus, more are needed to clarify how external factors can modify the epigenome in the SS and RA physiopathology contexts.

A lot of work is still to be done to improve the efficacy and safety of treatments against autoimmune diseases. However, the identification of the key epigenetic mechanisms and epigenetic switches controlling specific transcriptomic programs can provide new possibilities to elucidate the diagnosis of the wide array of autoimmune disorders. Therefore, research that specifically focuses on elucidating the epigenetic control in specific subpopulations of immune cells will provide new insights into the pathogenesis of autoimmune diseases and will open up new ways to develop novel therapeutic approaches that target epigenetic mechanisms, such as DNA methylation, histone PTMs, and regulating miRNAs expression. HDAC inhibitors (HDACi) are proposed as a therapeutic strategy for RA (Hsieh et al., 2014; Cantley et al., 2015; Oh et al., 2017; Angiolilli et al., 2018; Kim et al., 2018;). HDACi is a good therapeutic approaches because it helps to lower inflammation levels through vascular cell adhesion molecule-1 (VCAM), intercellular adhesion molecule-1 (ICAM), and E-selectin (ESEL) levels, which could contribute to immunosuppression (Inoue et al., 2006). Accordingly, the only clinical trial that has been tested is givinostat for juvenile idiopathic arthritis. Yet despite the benefits of this HDAC inhibitor, after a 12-week safety and efficacy assay (NCT00570661) (Vojinovic et al., 2011), a dose-finding study showed lack of efficacy (NCT0155745), which suggests that it should be used in conjunction with other treatments to improve the effect on patients. Moreover, more efforts should be made to further clarify the special contribution of epigenetics to the etiology of these immune-related disorders and how epigenetic-based drugs can increase the therapeutic options for RA and SS.

## Author Contributions

Bibliography compilation and analysis: JG-G, MS-C, JI-C, RO-V, and FP. Manuscript drafting: JG-G, MS-C, JI-C, RO-V, and FP. Review of manuscript content: MS-C and JI-C. Approval of the final version of the manuscript: all the authors.

## Funding

This work was supported by FIS (PI12/02263, PI16/01031, and PI16/01036) from ISCIII and was co-financed by European Regional Development Founds (ERDF) and ACCI2014 (CIBERer-ISCIII). JG-G is supported by the Spanish Ministry of Economy and Competitiveness and by the Instituto de Salud Carlos III through CIBERer (Biomedical Network Research Center for Rare Diseases and INGENIO2010).

## Conflict of Interest

The authors declare that the research was conducted in the absence of any commercial or financial relationships that could be construed as a potential conflict of interest.

## Abbreviations

ChIP, Chromatin Immunoprecipitation; CLP, Common Lymphoid Progenitors; DMRs, Differentially Methylated Regions; DNMTs, DNA Methyltransferases; HSCs, Hematopoietic Stem Cells; HDAC, Histone Deacetylases; IFN, Interferon; MHC, Major histocompatibility complex; PBMCs, Peripheral Blood Mononuclear Cells; PTMs, Post-Translational Modifications; ROS, Reactive Oxygen Species; TREG, Regulatory T-Cells; RA, Rheumatoid Arthritis; RASFs, Rheumatoid Arthritis Synovial Fibroblasts; SGECs, Salivary Gland Epithelial Cells; STAT, Signal Transducer and Activator of Transcription; SNPs, Single Nucleotide Polymorphism; SS, Sjögren Syndrome; miRNA, Small noncoding RNA.
